# *In vitro* replicative fitness of early Transmitted founder HIV-1 variants and sensitivity to Interferon alpha

**DOI:** 10.1038/s41598-020-59596-x

**Published:** 2020-02-17

**Authors:** Manickam Ashok kumar, Aanand Sonawane, Maike Sperk, Srikanth P. Tripathy, Ujjwal Neogi, Luke Elizabeth Hanna

**Affiliations:** 1 Department of HIV/AIDS, National Institute for Research in Tuberculosis, Chennai, India; 2 Division of Clinical Microbiology, Department of Laboratory Medicine, Karolinska Institute, Stockholm, Sweden; 3 University of Madras, Chennai, India; 4 Department of Microbiology and Immunology, Univeristy of Missouri, Columbia, MO 65211 USA

**Keywords:** Interferons, HIV infections

## Abstract

Type I interferons, particularly interferon-alpha (IFN-α), play a vital role in the host's anti-viral defenses by interfering with viral replication. However, the virus rapidly evolves to exploit the IFN-α response for its replication, spread, and pathogenic function. In this study, we attempted to determine IFN-α susceptibility and productivity of infectious transmitted/founder (TF) (n = 8) and non-transmitted (NT) viruses (n = 8) derived from HIV-1 infected infants. Independent experiments were carried out to determine IFN-α resistance, replication fitness, and viral productivity in CD4^+^ T cells over a short period. *In vitro* studies showed that TF viruses were resistant to IFN-α during the very near moment of transmission, but in the subsequent time points, they became susceptible to IFN-α. We did not observe much difference in replicative fitness of the TF viruses in cultures treated with and without IFN-α, but the difference was significant in the case of NT viruses obtained from the same individual. Despite increased susceptibility to IFN-α, NT viruses produced more viral particles than TF viruses. Similar results were also obtained in cultures treated with maraviroc (MVC). The study identified unique characteristics of TF viruses thus prompting further investigation into virus-host interaction occurring during the early stages of HIV infection.

## Introduction

Despite substantial progress in HIV treatment that has significantly reduced the risk of HIV-1 disease progression, the number of new infections has continued to increase significantly in the recent years^1^. Currently available antiretroviral drugs suppress viremia and stabilize CD4^+^ T cell count by preventing the virus replication cycle^2^. Concurrently, the virus evolves over some time by incorporating mutations to escape drug or humoral immune pressure and ensure its survival and fitness^3^. Understanding the key events occurring during acute HIV-1 infection, that play a key role in determining the course of the disease^4^, is therefore crucial for identification of an effective vaccine strategy that can protect against HIV-1 or other alternative prevention approaches^1^. Further, the phenotypic traits of infectious viral variants associated with enhanced mucosal transmission need to be identified and characterized^5^.

Disease progression in HIV-1 infected individuals varies in different stages of HIV infection. The different stages of HIV-1 infection are classified as primary/acute, asymptomatic/latent, and later/end-stage disease (AIDS). Acute infection is often associated with very high viral load^6^ and rapid depletion of CD4^+^ T cells^4^, while chronic HIV-1 infection is characterized by higher genotypic and phenotypic diversity^7^. Identifying the mechanisms involved in the interaction between the virus and the host during the acute phase of infection can provide a critical opportunity to prevent transmission of HIV infection.

It is believed that productive clinical HIV-1 infection is usually initiated by a single viral variant called the transmitted founder (TF) virus^7^. In general, these viruses are defined to exhibit chemokine receptor 5 (CCR5) tropism, target CD4^+^ T cells, possess shorter variable regions with fewer potential N-linked glycosylation sites (PNGs), enriched HIV-1 envelope (Env) content, enhanced cell-free infectivity and improved dendritic cell interaction, and are relatively more resistant to the antiviral effects of interferon-alpha (IFN-α)^8^. Targeting the functional envelopes responsible for the acquisition of clinical HIV-1 infection^9^ can block the spread of the virus^10^. Within a few weeks of natural HIV infection, most patients develop envelope-specific autologous neutralizing antibodies, but these antibodies fail to neutralize heterologous isolates^11^.

At the time of the establishment of infection, plasmacytoid dendritic cells (pDCs) have been shown to accumulate rapidly. These cells secrete cytokines and chemokines, particularly type I interferons (IFNs). The role of type I IFNs during HIV and SIV infections remains uncertain, with contradictory explanations^12^. In HIV-1 infection, IFN-α controls virus replication by several mechanisms including the stimulation of intrinsic restriction factors and induction of innate and adaptive immune responses^13^. However, the initial innate immune response elicited during the event of transmission is ineffective at suppressing SIV infection, and there is enhanced early viral replication^14^. IFN-α levels in individuals infected with HIV-1 have been shown to correlate with pathogenic immune activation^15^ and initiation of apoptosis in CD4^+^ T cells^12^. Also, interferons stimulate the expression of several genes, collectively known as interferon-stimulated genes (ISGs) that control pathogenic infections^16^. It was earlier reported that IFN-α alone did not change the rate of induced cell death, but in combination with interferon gamma (IFN-γ), synergistically increased induced cell death^17^. An enriched knowledge on the precise molecular and biological composition of TF viruses and their interaction with the host immune system is essential for identifying effective means of immunization against HIV infection.

## Results

### Definition of TF and NT variants

In general, the term Transmitted founder (TF) virus refers to a single^18–20^ or very few^21^ viral variant(s), that establish productive infection within a host. This definition is based on certain unique genetic and phenotypic properties such as the presence of fewer PNGs and shorter variable loops (V1V2)^7,22^. Studies have shown that shorter V1V2 loops and fewer PNGs are associated with enhanced sensitivity to neutralizing antibodies^7,22,23^. In addition, a consistent pattern of CCR5 co-receptor usage, greater resistance to fusion inhibitors^24^, increased infectivity and/or faster replication rate^25^ and resistance to IFN-α^26^ have also been reported as unique features of TF variants.

On the other hand, non-transmitted (NT) variants constitute a genetically diverse population of HIV-1 isolates that are sensitive to maternal antibodies^27^. Studies have shown that the viral variants in the transmitting partner were different from the transmitted founder virus in the newly infected partner^25^. In this study, we identified TF and NT variants by comparing the genetic identity between the different infectious clones generated from the same individual^28^ as well as the HIV-1 env DNA isolated from peripheral blood mononuclear cells (PBMC)^29^ of the infant. TF viruses and the provirus in the PBMCs are reported to have a very close genetic signature (shorter variable regions and fewer PNGs). Non-TF viruses have longer variable regions, greater number of PNGs and show increased sensitivity to neutralizing antibodies. The phenotypic differences between the two virus types described in this article have been reported earlier by our group^28^.

### Characteristics of chimeric viruses

All phenotypic assays were performed using 293 T cell-derived viral stocks from patient-derived gp120 cloned into pMN-K7-Luc-IRESs-NefΔgp120. Based on genotypic and phenotypic assays, the chimeric viruses were identified as either TF or NT viruses. The TF and NT chimeric viruses were independent viruses obtained from the same individual infected recently through MTCT. All the chimeric viruses were phenotyped as subtype C and were found to be CCR5 tropic, but few of the TF viruses used the CXCR6 co-receptor in addition to CCR5 for infection. Majority of the TF and NT viruses that were reported previously by others were derived from chronically infected individuals rather than from recently infected individuals. However, in this study, we derived all the chimeric viruses from recently infected individuals.

The chimeric viruses were normalized to 50% Tissue Culture Infective Dose (TCID_50_) by measuring luciferase expression in terms of relative light units (RLU) in TZM-bl cells. We used TCID_50_ for normalization of viral stocks because this value directly correlates with the absolute number of infectious viral particles, whereas quantification of p24 antigen or RT activity gives the total absolute number of viral particles that would include both infectious and non-infectious particles. Hence, normalization using p24 antigen or RT activity for the determination of MOI would not be accurate.

### Percentage of residual infection

We determined the saturation point of maraviroc (MVC) on the recombinant viruses by treating them with varying concentrations of 10-fold serial dilutions starting from 1 × 10^4^ nM and going down to 1 × 10^−3^ nM in a single-round infection assay in TZM-bl and GHOST (3) CCR5+ (Hi-5) cells. The EC_50_ values were found to be higher for TF viruses than for NT viruses when tested in TZM-bl cells; however, there was not a much difference seen in the GHOST (3) CCR5+ cells. To derive the saturation point, the percentage inhibition was calculated for each MVC concentration as the RLU measured 48 hours post-infection in both TZM-bl and CCR5 cells, and the percentage inhibition in the absence of MVC set to 100%.

**Figure 1:**
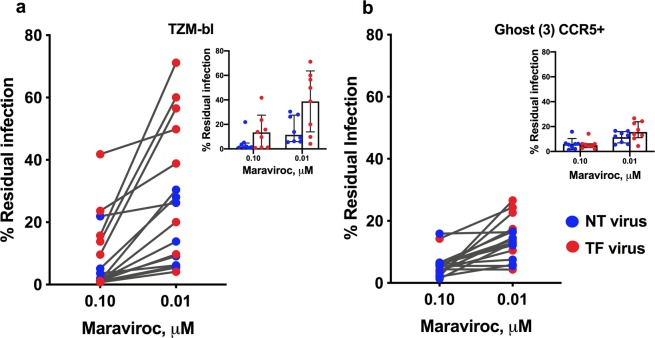
Percentage of residual infection of TF and NT viruses in TZM-bl cells (**a**) and GHOST (3) CCR5+ cells (**b**) treated with MVC at saturating concentrations. MVC saturation was confirmed by measuring residual infection between two concentrations where there was no difference in inhibition. No statistically significant difference in virus inhibition was observed between 0.1 and 0.01 μM MVC tested in GHOST (3) CCR5+ cells. The percentage of residual infection in two different cultures was plotted against the percentage of viral infection in the absence of MVC, which was set at 100%. Cells were seeded with different dilutions of MVC (10-fold dilutions starting from 1 × 10^4^ nM and going down to 1 × 10^−3^ nM) and incubated at 37 °C overnight before infection with chimeric virus.

It was observed that there was no increase in inhibition of viruses between 0.1 and 1 µM MVC concentration, indicating that MVC at or above 0.1 µM concentration did not inhibit the virus further. We defined the point at which there was no further inhibition of viral growth as the saturation point and determined percentage residual infection between the two concentrations. The average percentage residual infection by TF and NT viruses was found to be 38.8% and 15.7% respectively in TZM-bl cells (Fig. 1a), demonstrating greater than one-fold change (1.3) difference in percentage residual infection between the TF and NT viruses at 0.01 µM concentration. However, at 0.1 µM concentration, the difference was insignificant. Also, we did not observe a significant difference in the percentage of residual infection between TF and NT viruses tested in GHOST (3) CCR5+ cells (16.6% and 11.4% respectively) (Fig. 1b). Percentage inhibition of individual viruses is provided in Supplementary Fig. 1.

### Percentage IFN-α resistance in single round infection assay

**Figure 2:**
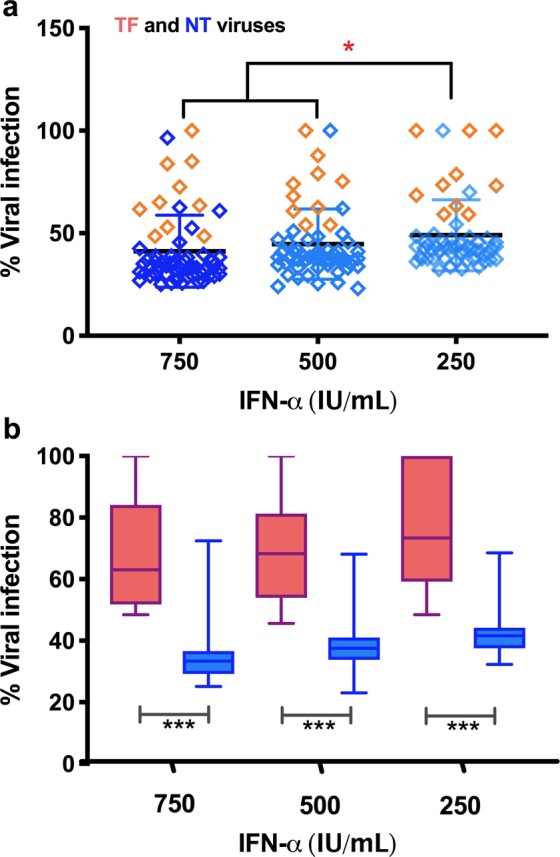
Single cycle replication fitness assay of TF and NT viruses in TZM-bl cells pretreated with different concentrations (0, 250, 500 and 750 IU/mL) of IFN-α. (**a**) Percentage infectivity by the chimeric viruses. Percentage of virus infection in cultures treated with IFN-α was plotted against the percentage of viral infection in the absence of IFN-α, which was set to 100%. (**b**) Percentage of IFN-α resistance of individual TF and NT viruses. Percentage resistance was determined as a ratio of viral infection in the presence and absence of IFN-α. P value was determined using two tailed t test. ***p < 0.001.

To determine the interferon set point to carry out the experiments in CD4^+^ primary T cells, we tested three different concentrations (250, 500 and 750 IU/mL) in TZM-bl cells with chimeric viruses. Virus expression was measured by the intensity of RLU for each IFN-α concentration after 48 hours of infection and plotted as the percentage of viral infection in the absence of IFN-α, which was set to 100%. The expression of each chimeric virus isolate was calculated based on RLU levels in normal condition. For few chimeric viruses which resulted in higher levels of viral expression in the presence of 250 IU/mL of IFN-α than in the absence of IFN-α, the percentage of expression was normalized to 100%. The average percentage of infection against different concentrations (250, 500 and 750 IU/mL) of IFN-α tested was found to be 49, 44.6 and 41.3% respectively (Fig. 2a).

The average percentage infection of chimeric viruses in the presence of 750 and 500 IU/mL of IFN-α was found to be nearly similar. However, the infection rate varied significantly between TF and NT viruses identified from the same individual (Fig. 2b). The infection rate at 750 IU/mL of IFN-α was found to be 67.1% for TF virus whereas, the infection rate for the corresponding NT virus was 34.2% with nearly one-fold (0.97) difference. However, the fold change was almost equal (0.85 and 0.87) at concentrations between 250 and 500 IU/mL with infection rates of 75.6 and 69.3% for TF and 41.8 and 37.8% for NT viruses, respectively. The infection rate differed significantly at all the three concentrations of IFN-α between TF and NT viruses.

### Transmitted founder viruses are relatively more resistant to IFN-α

**Figure 3:**
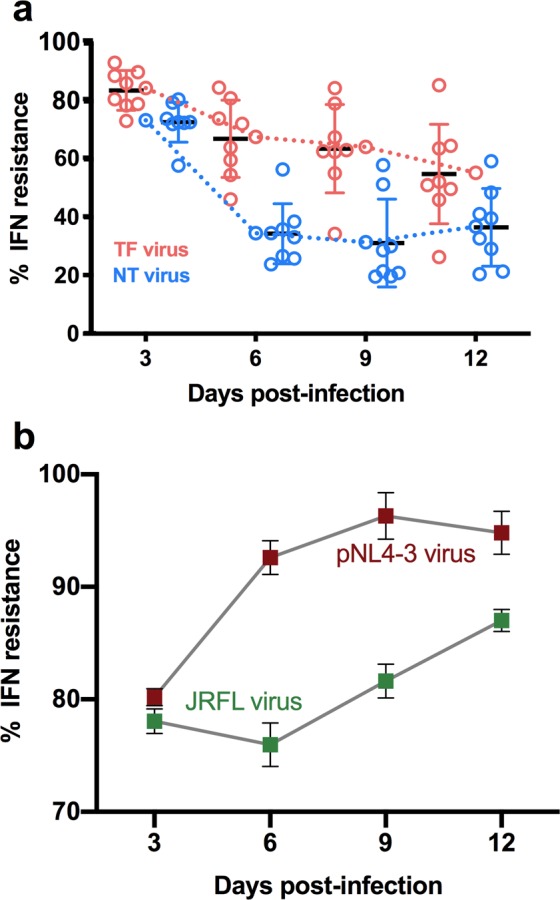
Percentage IFN-α resistance of chimeric viruses in CD4^+^ T cells. (**a**,**b**) The percentage resistance defined as the ratio of virus produced in the presence and absence of IFN-α is plotted for each virus (y axis) at different time points (days) post-infection (x axis). Percentage IFN-α resistance of chimeric viruses (**a**) and virus control (**b**) are represented with the colors mentioned in the figure.

Recent studies in subtype C HIV-1 infection showed that there was no significant difference in IFN-α activity on TF and NT viruses derived from the same individual^25^. On the other hand, in individuals infected with subtype B virus, the TF viruses were found to be relatively more resistant to IFN-α than NT viruses^30^. To substantiate the antiviral effect of IFN-α on subtype C chimeric viruses derived from recent vertical infection, we infected phytohemagglutinin (PHA) stimulated primary CD4^+^ T-cells with an equal amount of TF and NT viruses from the same individual by treating one half with IFN-α and the other half without IFN-α. p24 antigen release in cultures treated with and without IFN-α was quantified as an indirect measure of resistance to IFN-α for the TF and NT viruses. The percentage resistance at each time point for TF and NT viruses is presented in Fig. 3a. We noticed only a small difference in resistance between TF and NT viruses at the earliest time point post-infection (3^rd^ day). However, IFN-α resistance of TF viruses increased to nearly two-fold change than that of NT viruses at two consecutive time points (6^th^ and 9^th^ day). The percentage resistance of TF viruses declined significantly to 67 and 63.7% on the 6^th^ and 9^th^ day respectively from 84% on the 3^rd^ day. Subsequently, on day 12 it was observed that the NT viruses became resistant to IFN-α and that the TF viruses lost their resistance to IFN-α. The difference in resistance between the two groups of viruses was statistically significant at all time points except on day 3. At the same time, the percentage of resistance for pNL4-3 and JRFL viruses in culture supernatants at the earliest time point was around 80% (Fig. 3b). However, in the next consecutive time points, the percentage resistance of pNL4-3 virus increased significantly and then started decreasing at the latest time point (12^th^ day). In contrast, the JRFL virus responded slightly to IFN-α on the 6^th^ day and after that started gaining resistance to IFN-α.

### IFN-α resistance determined by the frequency of Gag-positive cells

**Figure 4:**
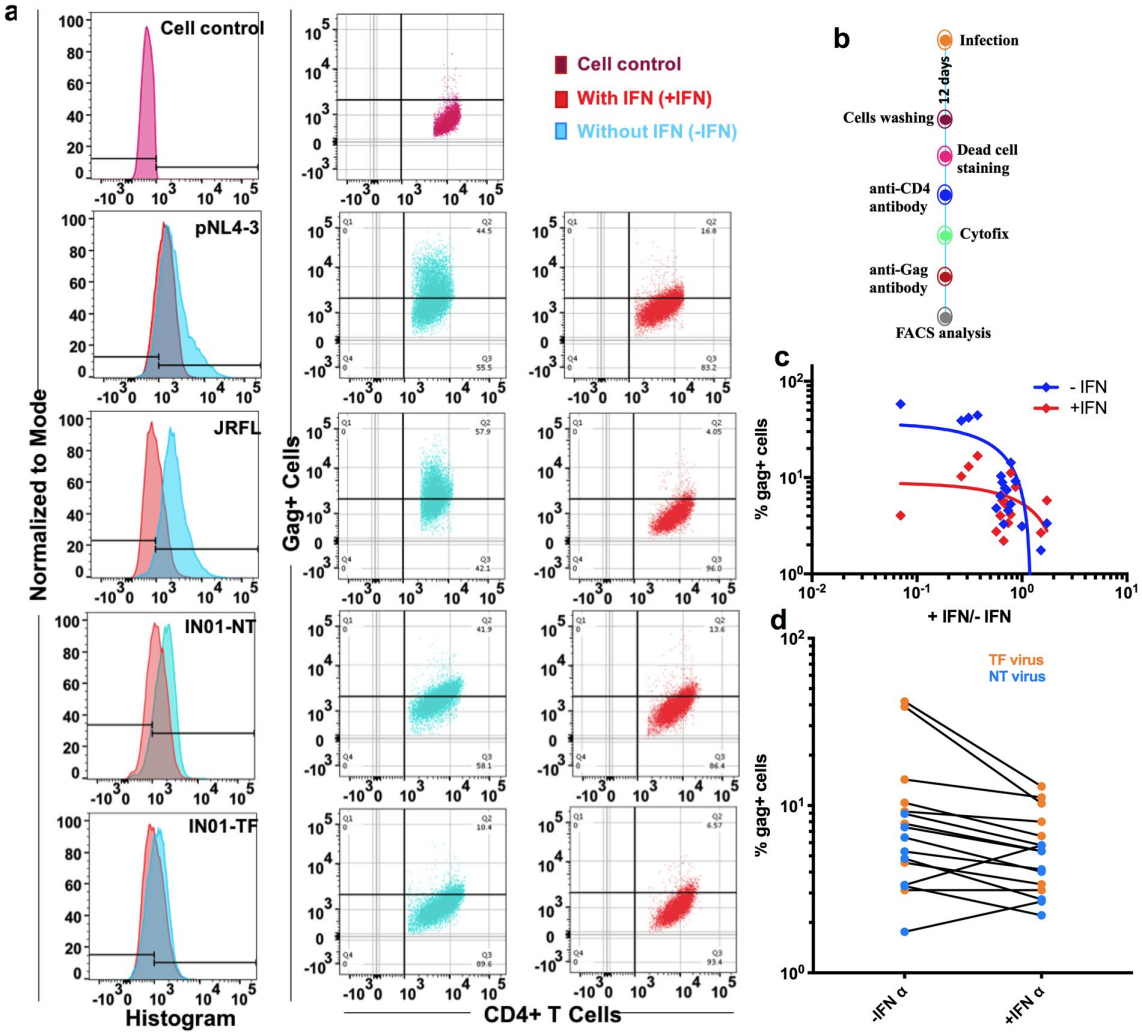
Gag-positive cells among CD4^+^ T cells (with and without IFN-α) infected with different viruses. Expression of Gag-positive cells was measured 12 days post-infection. (**a**) Gag-positive cells in TF and NT virus infected CD4^+^ T cell cultures pretreated with and without IFN-α. Purple colored histograms represent mock-infected cell control. Histograms coloured red and cyan represent Gag-positive cells for the TF and NT viruses in the presence and absence of IFN-α respectively. Dot blot represents Gag-positive cells in the absence (left) and presence of IFN-α. (**b**) Summarized work flow for the preparation of CD4^+^ T cells for FACS analysis. (**c**) Correlation between percentage of Gag-positive cells (y axis) with respect to ratio of Gag-positive cells in the absence and presence of IFN-α (x axis). (**d**) Percentage expression of Gag-positive cells in the absence and presence of IFN-α. The color code for TF and NT viruses is defined in the right panel.

In addition to the quantification of p24, we sorted the gag tagged CD4^+^ T cells 12 days post-infection with TF and NT viruses for the determination of IFN-α resistance. The presence of Gag-positive cells by flow cytometry was used as an indirect measure of infection of CD4^+^ T cells. The mock-infected CD4^+^ T cell culture was considered as cell control and was used to set the manual gate to calculate the percentage of virus-infected cells. Data obtained from FACS analysis are presented in Fig. 4. Non-viable cells were removed by manual gating based on live/dead cell staining. Figure 4a shows the expression of Gag-positive CD4^+^ T cells in cultures treated with and without IFN-α. The preparation of CD4^+^ T cells for FACS analysis is summarized in Fig. 4b. Taken together, the ratio of virus produced by the chimeric viruses in the presence and absence of IFN-α correlated significantly with the percentage of Gag-positive cells (P = 0.048) (Fig. 4c). Gag-positive CD4^+^ T cells were found to be higher in the TF virus group than in NT viruses, though the difference was not statistically significant (Fig. 4d). Notably, a few TF viruses replicated to significantly high titers in the absence of IFN-α than in the presence of IFN-α. These seemingly disparate findings in contrast to the previous findings^26^, could be either because the virus produced from the CD4^+^ T cells infected by TF virus became sensitive to IFN-α, or because CD4^+^ T cells infected by NT virus produce IFN-α resistant particles during the course of infection.

### Replication kinetics of chimeric viruses in the presence and absence of IFN-α

**Figure 5:**
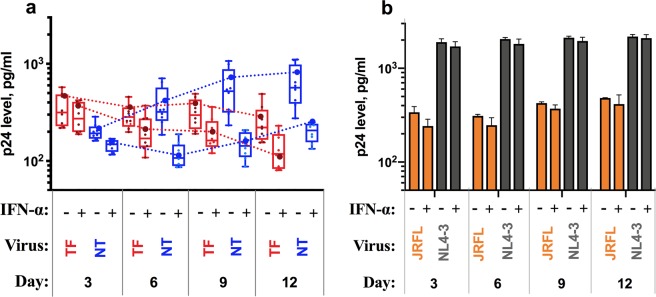
Replication kinetics of TF and NT viruses in CD4^+^ T cells in the presence and absence of IFN-α. (**a**) Viral replication in the presence and absence of IFN-α is plotted for each virus (y axis) at different time points (days) post-infection (x axis); red and blue color represent TF and NT virus in the presence and absence of IFN-α. (**b**) Viral replication of JRFL and NL4-3 viruses in the presence and absence of IFN-α at different time points (days) post-infection.

We next sought to investigate the replication kinetics of TF and NT viruses in primary CD4^+^ T cells. CD4^+^ T cells in the mucosa-associated lymphatic tissue are the predominant targets of HIV and are infected productively soon after the moment of transmission^26,31^. PHA-stimulated CD4^+^ T cells derived from healthy individuals were infected with an equal number of virus particles (based on the TCID_50_ value) and the replication kinetics of a subset of chimeric viral isolates (*n* *=* 16) along with tropism dependent and independent viral controls (JRFL and pNL4-3) were determined. The experiments were carried out in the presence and absence of IFN-α for 12 days, and p24 antigen was measured in culture supernatants collected every 72 hours. We then compared the p24 antigen released by TF and NT virus-infected CD4^+^ T cells cultured in two different conditions (with and without IFN-α) between single and multiple time points and the results are presented in Fig. 5a.

In the absence of IFN-α, the average total p24 released by TF viruses was comparatively lesser than that of NT viruses: 1229 pg/mL for TF viruses (interquartile range [IQR]: 767 to 1848 pg/mL) and 1785 pg/mL for NT viruses (IQR: 856 to 3159 pg/mL). p24 levels measured at the earliest time point in TF viruses (3^rd^ day post-infection) was not significantly different from that of NT viruses. The p24 level at this time point was 356 pg/mL (IQR: 219 to 575 pg/mL) for TF viruses and 202 pg/mL (IQR: 161.4 to 286 pg/mL) for NT viruses with a 0.82-fold change. However, between days 6 to 12, the p24 level decreased for TF viruses, as was reported previously^26^, when compared to NT viruses.

In contrast, the sum of the mean p24 levels of TF viruses was found to be 790 pg/mL (IQR: 510 to 1318 pg/mL), which was higher than that of NT viruses which was estimated as 614 pg/mL (IQR: 444 to 796 pg/mL) in cultures treated with IFN-α. The average p24 level at three different time points (day 3, 6 and 9) for TF viruses (251, 206 and 198 pg/mL) was significantly higher than for the NT viruses (146, 119 and 149 pg/mL) respectively. However, the amount of p24 determined on day 12 was found to be half-fold lower for TF viruses when compared to NT viruses (135 vs 200 pg/mL). The p24 level at different time points and different culture conditions for all the viral isolates tested are provided in Supplementary Fig. 2.

When compared to JRFL and pNL4-3 which served as controls (Fig. 5b), the TF viruses showed similar or about one-fold lower p24 levels in supernatants collected at different time points from cultures with and without IFN-α. In contrast, the NT viruses showed increased levels of p24 in all supernatants harvested at different time points, except on day 3, in cultures without IFN-α than JRFL. But, it was reversed in cultures with IFN-α. In both the conditions, the p24 level in TF and NT viruses was approximately one-fold lower than that of the pNL4-3 virus. When the virus controls were compared with each other, the p24 level of pNL4-3 was found to be one-fold higher than that of JRFL in supernatants harvested from both culture conditions and at all time points.

### Viral release and productivity

**Figure 6:**
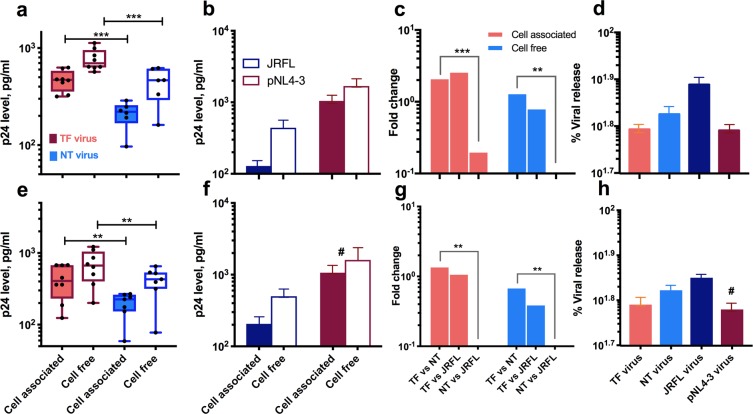
Viral particle release and productivity of TF and NT chimeric viruses in CD4^+^ T cells treated with IFN-α (**a**–**d**) and MVC (**e**–**h**). (**a**,**e**) cell free and cell associated viral particles 9 days post-infection in CD4^+^ T cells infected with TF and NT viruses represented as pg/mL of p24. (**b**,**f**) Cell-free and cell-associated viral release 9 days post-infection with JRFL and pNL4-3 virus. (**c**,**g**) Fold change difference in viral release by TF, NT, JRFL viruses. Fold change was determined based on the calculation defined in the statistical analysis section. (**d**,**h**) percentage viral release/productivity by the viruses in culture under the influence of IFN-α and MVC. The percentage viral release is determined based on the calculation mentioned in the Materials and Methods section. All the viral groups are color coded and defined within the figure. ^#^pNL4-3 virus infected culture was treated with AMD3100, CXCR4 antagonist. Log10 scale with power of 10 value was used for y axis. **P < 0.01; ***P < 0.001.

Encouraged by the previous findings, we went one step further to quantify the production of viral particles from CD4^+^ T cells infected with TF and NT viruses in cultures treated separately with IFN-α and MVC before infection. Newer studies have provided evidence that CD4^+^ T cells infected with TF viruses release comparatively vast numbers of cell-free viruses than NT virus-infected cells^26^. We measured the amounts of cell-associated and cell-free p24 antigen nine days post-infection and used these values to determine the productivity of TF and NT viruses. As expected, total (both cell-free and cell-associated) amount of p24 was found to be significantly higher with 1.3 and 0.9 fold increase for TF viruses than for NT viruses in cultures treated with IFN-α and MVC respectively. Also, there was no significant difference between cultures infected with TF viruses as compared to JRFL virus in both the conditions, but the fold change was 1.2 and 1.4 lower when compared to the pNL4-3 virus in respective conditions. The amount of p24 antigen produced and fold change values are presented in Fig. 6a–c for IFN-α and 6e-g for MVC.

However, it was surprising to note that the ability to produce viral particles in IFN-α and MVC selected cultures infected with TF viruses was lower than for cultures infected with NT viruses (Fig. 6d,h). This difference though was not statistically significant. The productivity of p24 in cultures infected with the chimeric viruses and control viruses (JRFL and pNL4-3) were comparable with no statistical significance. The average productivity of TF and NT viruses was around 62 and 67% respectively in both IFN-α and MVC-treated cultures, whereas the productivity with JRFL and pNL4-3 was 77 and 62% respectively in IFN-α-selected and 71 and 60% in MVC-selected cultures respectively.

## Discussion

Among the repertoire of viral variants that are transmitted from an HIV-infected individual during the transmission event, the subset of virus(es) that initiate successful infection (TF viruses) have not been fully characterized yet. Improved knowledge on virological characteristics of the TF viruses will provide a major impetus for vaccine design. In this study, we generated several infectious chimeric virus clones from eight HIV-infected infants during the very early stages of infection and classified them as TF and NT viruses based on their genetic and phenotypic properties. TF viruses had short variable regions with a fewer number of glycosylation sites in their envelope and possessed unique phenotypic properties such as higher infectivity titer, differential co-receptor usage, reduced sensitivity to neutralizing antibodies and the co-receptor antagonist, MVC, as reported in one of our earlier studies^28^. NT viruses failed to exhibit any of the characteristics mentioned above.

In the present study, we aimed to investigate the percentage residual infection by the chimeric viruses against MVC, a CCR5 antagonist, which potentially inhibits HIV infection particularly that of R5 strains, as proven in experimental and clinical studies^32,33^. We treated Ghost (3) CCR5+ and TZM-bl cells with different concentrations of MVC and then infected them with TF and NT viruses. We found that the saturating concentration of MVC (concentration above which there is no difference in inhibition) ranged between 0.1 and 1 μM in TZM-bl cells. However, in CCR5+ cells the saturation point was reached at 0.01 μM concentration of MVC. Hence, we determined the percentage residual infection of chimeric viruses at 0.01 μM MVC in both CCR5+ and TZM-bl cells. While we found no difference in response to saturating concentrations of MVC between TF and NT viruses in CCR5+ cells, we observed a statistically significant difference between TF and NT virus isolates in TZM-bl cells that are highly sensitive to HIV infection and express large amounts of CD4 and CCR5 co-receptor. In contrast, a previous study had reported that in the presence of saturating concentration of MVC, the infection was more likely to be mediated by chronic envs than acute envs^32^. Preexisting resistance to MVC observed in TF viruses could be explained as a result of adaptive variations in gp120 that abrogates binding of the active form of the CCR5 antagonist^34^, or the possible use of alternate co-receptors for infection by the viral isolates present in the early stages of infection^35,36^.

In addition, we sought to examine the sensitivity/resistance of the viruses to the anti-viral factor IFN-α which is produced by the host at the time of infection. There is some evidence to indicate that TF viruses possess an excess measure of resistance to IFN-α as compared to chronic viruses^7,26^. We tested the sensitivity/resistance of different TF and NT viruses derived from the same individual to IFN-α, intending to understand differences in the virus-host interaction between the diverse circulating virus isolates within a given individual, and the evolutionary mechanisms operating in the virus to counter the host immune pressure. IFN-α resistance in TF viruses to the extent of about one-fold change more than NT viruses was observed in the TZM-bl cells.

Since single-round infectivity assay to determine the potential role of IFN-α could not provide useful information on viral resistance, replication, and productivity of TF and NT viruses, we performed a multiple round infection assay in primary CD4^+^ T cells in the presence and absence of IFN-α to compare the resistance, replication kinetics and productivity of TF and NT viruses over a period of time up to 12 days. Quantification of HIV-1 p24 antigen from the supernatants collected from cultures treated with and without IFN-α at different time points and the estimation of Gag-positive cells by flow cytometry revealed that the TF viruses were moderately more resistant to IFN-α than NT viruses, as was also reported in earlier studies^7,8,26^. But over some time, the level of resistance decreased in TF viruses. A very recent study also showed that HIV-1 TF viruses are more resistant to IFN-induced transmembrane (IFITM) proteins^37^. Similar to our findings, Foster *et al*. (2016) also reported that the TF virus acquires sensitivity to IFN-α over a prolonged period^38^. Another study, in contrast, reported that resistance to IFN-α is not a determinant for TF viruses for infection establishment^20^.

The increased sensitivity to IFN-α seems to correlate with the acquisition of escape mutations by the *env* since the time of the early autologous neutralization response^39^. IFN-α sensitivity was previously demonstrated in CD4^+^ T cells of patients chronically infected with HIV-1^8^. In contrast, in another *in vivo* study, ten days of treatment with IFN-α did not result in a significant reduction in p24 levels as compared to mock-treated model^13^. Flow cytometric analysis revealed that the percentage of Gag-positive cells in TF virus-infected CD4^+^ T cells were only slightly higher than in NT virus-infected CD4^+^ T cells. Further, interferon resistance was found to be higher in the X4-tropic control virus during the early stages of infection than R5-tropic control virus. As described in an earlier study, we also observed that TF viruses were few folds less infectious than the lab-adapted strains, pNL4-3^19^ and JRFL. During infection, it was noticed that the difference in resistance reduced, and the Gag-positive cell count was identified to be nearly equal in TF and NT virus-infected cell cultures on the 12^th^ day of infection. However, the enhanced resistance to interferon/higher infectivity titer exhibited by the lab adapted stains was higher than that of TF viruses^19^. IFN-α upregulates the expression of the several well-known HIV-1 and SIV restriction factors such as TRIM5α (tripartite motif-containing protein 5α), APOBEC3G (apolipoprotein B mRNA-editing enzyme, catalytic polypeptide-like 3G), tetherin and SAMHD1 (SAM and HD domain-containing protein 1) in addition to HIV-1 resistance factors such as myxovirus resistance 2 (MX2), schlafen 11 (SLFN11), IFN-induced transmembrane (IFITM) proteins^38^, BCL-G^40^ and Cytidine/Uridine Monophosphate Kinase 2 (CMPK2)^40,41^, which constrain HIV-1 replication at different steps. Several studies have reported that early release of IFN-α suppresses the viral load during the acute phase of HIV-1 infection. In contrast, over a period of time after infection, researchers discovered a positive correlation between plasma IFN-α level and disease progression^42^.

In the absence of IFN-α, viral replication kinetics at the earliest time point (day 3) was found to be 0.82 fold higher for TF virus, but in the subsequent time points, it was lower than that of NT virus. Alternatively, the viral replication kinetics of TF viruses in the presence of IFN-α was found to be more than half-fold higher during the first two time points after infection. Surprisingly, the replication kinetics was 0.33 fold higher for TF viruses, which is half fold lower than at the previous time point and 0.32 fold higher in NT viruses in the next consecutive time points respectively. In aggregate, these observations strongly support the fact that TF viruses as reported previously possess unique features that give them the advantage of successfully establishing infection, but shortly lose their resistance to IFN-α. A recent study on epidemiologically linked pairs failed to infer higher replication fitness and interferon resistance of transmitted viral variants than non-transmitted variants within an individual^25^. This study showed that the NT viruses also had equal fitness to replicate at higher titers during infection in drug naïve individuals.

Furthermore, it has been reported that TF viruses with shorter and less glycosylated envelopes, increased their envelope length, glycosylation sites, and V3 charge during the course of infection^43^. This could contribute to the increased sensitivity of the virus to IFN-α and significantly reduce viral replication kinetics in different primary cell culture systems. It was an extra measure of evidence that chimeric viruses with envelopes obtained from recently infected individuals soon after the moment of transmission exhibit significantly lower levels of replication than viruses circulating in chronically infected individuals^43^.

With this interesting finding, we additionally performed an independent experiment to determine the productivity of TF and NT viruses under the influence of IFN-α, by measuring cell-free and cell-associated viral particles nine days post-infection. We did find a 1.6-fold change and 1.2-fold change higher amount of cell-associated and cell-free viruses in cultures infected with TF viruses than with NT viruses respectively. This would appear that TF viruses carry functional envelope spikes in excess that mediates enhanced infection in primary CD4^+^ T cells than chronic viruses^7^, which is in agreement with previous studies which reported that the TF viruses release an excess amount of viral particles than chronic viruses^26,44^.

However, it was surprising to note that the percentage of viral production, that is, the ratio of cell-free and total virus (sum of the cell-free and cell-associated virus) was found to be higher in NT viruses than in TF viruses. This was in contrast to the findings of Iyer *et al*.^26^ which suggested that TF viruses replicate with higher titer than NT viruses. But the observed difference in productivity was merely 4%. Additionally, we undertook an experiment with MVC to counter-check the productivity of TF viruses in primary CD4^+^ T cells. Contrary to what was expected, the productivity was found to be significantly lower for TF viruses than NT viruses, even though the cell-free viral particles measured at various time points was found to be higher for TF viruses. In support of our data, a recent study has reported that MVC-sens/res Envs delayed replication kinetics of HIV-1 in the presence, but not in the absence of MVC^45^. It remains unclear how the productivity of TF viruses was lower in spite of the higher amounts of the cell-associated virus as compared to NT viruses. However, earlier investigations on the viral genome have shown rapid acquisition of escape mutations by HIV-1 associated with a gradual drop in viral load immediately after acute infection^46^. These data suggest that the productivity of TF and NT viruses as well as its replication kinetics within an individual may not be determined over a period of time concerning the immune response and escape for the survival. This study has a limitation that needs to be mentioned. As described previously^28^, the backbone used for cloning gp120 region of HIV-1 was that of subtype B. The viruses produced from these clones were recombinants of both subtype C and B. The subtype B gene could have differentially influenced infection. However, the driving force was similar for both TF and NT viruses in the current study.

To summarize, there are very limited studies that have characterized in detail the differential co-receptor usage^7^, replicative fitness^25^, interferon resistance^20,25,30^ and viral productivity^26^ of TF and NT viruses within an individual or between linked transmission pairs. In a previous study, we demonstrated differential co-receptor usage of TF viruses^28^. For the very first time, we have reported on the interferon resistance and fitness of TF and NT viruses within an individual here. TF viruses posseses enhanced interferon resistance at the very early time point, but the resistance diminishes with time. In contrast, NT viruses become resistant over a period of time. In addition, our study demonstrates that NT viruses are more productive than TF viruses. This collective finding suggests that the unique features of TF viruses may play an important role in HIV-1 transmission, but may not be sufficient to explain the differential success of TF viruses in establishing infection and determine HIV-1 disease progression and fitness. Additional investigations are therefore required to determine the role of IFN-α and its interference in signaling pathways in acute HIV-1 infection.

## Materials and Methods

### Ethics statement

The study and all experimental protocols were approved by the Institutional Ethics Committee of the National Institute for Research in Tuberculosis (formerly known as Tuberculosis Research Centre ICMR; TRC-IEC 2009009) and all methods were performed in accordance with relevant guidelines and regulations. Sample collection was done after obtaining written informed consent from the parents of the study participants.

### Description of plasmids/chimeric viruses

The development of infectious gp120-pMN-K7-Luc-IRESs-NefΔgp120 clones used in this study has been described previously^28^. Briefly, RNA was isolated from the plasma of eight infants who acquired HIV infection through mother-to-child transmission (MTCT), and converted into cDNA. The gp120 gene was amplified and cloned into pMN-K7-Luc-IRESs-NefΔgp120 by restriction digestion and ligation. Chimeric viruses were produced by transfecting 293 T cells. Virus supernatants were clarified by centrifugation, aliquoted, and stored at −80 °C until use.

The plasmid, pMN-K7-Luc-IRESs-NefΔgp120, was kindly gifted by Thomas Klimkait (University of Basel, Basel, Switzerland). HIV-1 NL4-3 Infectious Molecular Clone (herein, pNL4-3) (Lot number: 160159) and HIV Gag-iGFP-JRFL (herein, JRFL) (Lot number, 150117) were obtained from the NIH AIDS Reagent Program, Division of AIDS, NIAID, NIH. As per the data sheet, virus produced from JRFL is infectious in single round infection assays but is defective in multi-round replication in most cells. However, a newer and earlier study observed that JRFL has significantly higher cell-to-cell transfer efficiency, with donor nucleofected Jurkat cells, MT4R5 cells^19^, and primary CD4^+^ T cells^47^. We, therefore, used JRFL as a R5 tropic control for both single and multiple round infection assays in primary CD4^+^ T cells along with pNL4-3 as X4-tropic control.

### Cell lines

293 T cells (ATCC^®^ CRL-3216™), TZM-bl indicator cells (Lot number 150078), and GHOST (3) CCR5+ (Hi-5) cells expressing CD4 and higher levels of CCR5 (Lot number 150236), were maintained in Dulbecco's Modified Eagle medium (DMEM) with 10% (v/v) fetal bovine serum (FBS) (Sigma-Aldrich) and 1% penicillin-streptomycin. Primary CD4^+^ T cells isolated from PBMC of healthy individuals were maintained in Roswell Park Memorial Institute (RPMI) 1640 medium supplemented with 10% (v/v) FBS (Sigma-Aldrich), 1% penicillin-streptomycin and 20 U/mL recombinant interleukin-2 (IL-2). The CD4^+^ T cells used in the present study were isolated from a single healthy donor, and the purity was checked by flow cytometry. The cell lines, TZM-bl from John C. Kappes, Xiaoyun Wu, and Tranzyme Inc., and GHOST (3) CCR5+ cells (Hi-5) from Vineet N. Kewal Ramani and Dan R. Littman were obtained through the NIH AIDS Reagent Program, Division of AIDS, NIAID, NIH.

### Determination of saturation point for maraviroc

TZM-bl and GHOST (3) CCR5+ (Hi-5) cell lines were employed to assess the sensitivity of chimeric viruses to the CCR5 antagonist, maraviroc. Briefly, 10^4^ TZM-bl and Hi-5 cells were plated in each well of a 96-well plate 24 hours before infection. Cells were incubated with different dilutions of MVC (10-fold dilutions starting from 10 μM up to 10^−3^ nM) overnight at 37 °C before inoculation with chimeric viruses. Virus infected cells cultured in the absence of MVC served as negative control. At 48 hours post-infection, cells were lysed with 1X passive lysis buffer (Bright-Glo luciferase assay system) and luciferase expression was measured using an Infinite M200Pro plate reader (Tecan, Switzerland) as the number of relative light units (RLU). All conditions were tested in triplicate in each of at least two independent experiments.

### Single round virus replication kinetics assay

For single cycle infectivity assay, virus stocks normalized by TCID_50_ and further diluted to 0.01 MOI were used to infect TZM-bl cells (1 × 10^4^ cells/well) in triplicates in the presence of DEAE dextran (10 μg/mL). Cells were pre-incubated for 2 hours with different concentrations (0, 250, 500 and 750 IU/mL) of IFN-α at 37 °C before infection with chimeric viruses. 48 hours after infection, luciferase activity was measured using the Bright-Glo luciferase assay system (Promega).

### Multiple round virus replication kinetics assay

CD4^+^ T cells were isolated from buffy coats by depleting CD8^+^ T cells and stimulated with PHA (20 μg/mL) in the presence of 20 U/mL IL-2 in RPMI medium containing 10% FBS at a concentration of 1 × 10^6^ cells per milliliter. Three days post-stimulation, cells were washed and transferred to a 96-well V-bottom plate, resuspended at a concentration of 2 × 10^6^ cells per well, and infected with an equivalent amount of each virus (based on TCID_50_) in 250 μl of culture medium. Plates were then spinoculated at 800 × g for 2 hours at 4 °C followed by overnight incubation at 37 °C in a CO_2_ incubator. On the next day, cells were washed three times with 1 mL PBS and divided into two: one-half of the cells were cultured in T-cell medium alone, and the other half was cultured in medium supplemented with 500 IU/mL recombinant IFN-α (PBL Assay Science) in a 48-well plate; 200 μL of medium was sampled, and fresh medium was replaced, every 3 days. On day 6, 800 μL of the medium was removed and replenished with fresh medium containing 500 IU/mL IFN-α. Cells were propagated in medium containing 20 U/mL IL-2 in the presence and absence of 500 IU/mL IFN-α for up to 12 days.

On day 12, cells and supernatant were separated by centrifugation. The supernatants collected at different points of time (3, 6, 9 and 12 days) were stored at −80 °C until use. Supernatants were subjected to p24 antigen measurement using the Alliance HIV-1 P24 antigen ELISA Kit (PerkinElmer Inc.) following the manufacturer's protocol. Cells collected on day 12 were analyzed by flow cytometry.

### Expression of Gag-positive cells

Cells collected on day 12 post-infection were washed three times in PBS and incubated with 1 μL of 1:1,000 dilution LIVE/DEAD™ Fixable Aqua Dead Cell Stain (Invitrogen) in DMSO. Cells were incubated for 30 minutes at 4 °C with anti-CD4 antibody (APC/Cy7 anti-human CD4 antibody; BioLegend), washed, permeabilized using the Cytofix/Cytoperm Kit (BD Biosciences) according to the manufacturer's instructions, and incubated with anti-Gag antibody (HIV-1 core antigen, KC-57 RD-1; Beckman-Coulter) for 1 hour at 4 °C. Cells were analyzed on a BD FACSVerse^TM^ instrument using BD FACSuite^TM^ software version 1.0. Gag-positive cells were expressed as a percentage of all living cells. Mock-infected culture from the same donor was used to define baseline anti-Gag staining. Cell viability on day 12 was compared with that of mock-infected culture.

### Flow cytometric data analysis

Data were acquired with the BD FACSuite^TM^ software, version 1.0, and automatically compensated with the compensation matrix recorded within the sample files according to FCS 3.0 standards. Manual gating was performed in FlowJo v.10.2–10.3 (FlowJo, Inc) while blinded to sample identifier. The gating was set to 0 for Gag-positive cells in mock-infected CD4^+^ T cells to compare the infectivity by the chimeric virus in the presence and absence of IFN-α. Numeric values from FlowJo data analysis were exported to Prism 8.0 (GraphPad, US) for statistical analysis. Dead cells, debris, CD14+ cells, and CD19+ cells were excluded by manual gating. After clustering by manual gating, the percentage of infected cells was determined based on KC-57 RD-1 marker and the difference between IFN-α -selected, and non-selected cultures were measured.

### Quantification of virus release in the presence of interferon and maraviroc

CD4^+^ T cells treated with 500 IU/mL IFN-α and 10 µM MVC in two independent cultures were infected with TF and NT virus as described above. To quantify cell-associated and cell-free p24, cells and supernatants were harvested nine days post-infection by centrifugation at 1,200 rpm for 5 min, cells were lysed, and p24 antigen levels were quantified as described above. For each isolate, total p24 production was calculated by adding cell-free and cell-associated p24 levels. Percentage of virus release was determined by dividing the cell-free p24 by the total amount of p24 as described previously^48^.

### Statistical analysis

Values obtained from a panel of TF and NT chimeric viruses were compared using a one-way ANOVA test followed by multiple comparisons using GraphPad Prism, version 8.0 software. A nonparametric two-tailed t-test was performed for intra-pair comparison to compare the control and test groups. P values less than 0.05 were considered statistically significant. Fold-change value was calculated as the log_2_ value of the ratio between two groups. Fold-change threshold was set to compare the difference between the groups.

## Supplementary information

Supplementary information

## Data Availability

The authors confirm that the data supporting the findings of this study are available within the article and its supplementary materials.
