# Publisher Correction: *In vitro* replicative fitness of early Transmitted founder HIV-1 variants and sensitivity to Interferon alpha

**DOI:** 10.1038/s41598-020-68818-1

**Published:** 2020-08-10

**Authors:** Manickam Ashokkumar, Aanand Sonawane, Maike Sperk, Srikanth P. Tripathy, Ujjwal Neogi, Luke Elizabeth Hanna

**Affiliations:** 1 Department of HIV/AIDS, National Institute for Research in Tuberculosis, Chennai, India; 2 Division of Clinical Microbiology, Department of Laboratory Medicine, Karolinska Institute, Stockholm, Sweden; 3 University of Madras, Chennai, India; 4 Department of Molecular Microbiology and Immunology, University of Missouri, Columbia, MO 65211 USA

Correction to: *Scientific Reports*10.1038/s41598-020-59596-x, published online 17 February 2020

The original version of this Article contained a typographical error in the spelling of the author Manickam Ashokkumar, which was incorrectly given as Manickam Ashok kumar.

In addition, there was an error in Affiliation 4, which was incorrectly given as ‘Department of Microbiology and Immunology, Univeristy of Missouri, Columbia, MO, 65211, USA’. The correct affiliation is listed below:

Department of Molecular Microbiology and Immunology, University of Missouri, Columbia, MO 65211, USA.

Furthermore, the Supplementary Information file omitted an affiliation for Aanand Sonawanne, which is now listed as Affiliation 3.

These errors have now been corrected in the PDF and HTML versions of the Article, and in the accompanying Supplementary Information.

