# Supplementary material for: In vitro replicative fitness of early Transmitted founder HIV-1 variants and sensitivity to Interferon alpha
Source: Sci Rep. 2020 Feb 17;10:2747. doi: 10.1038/s41598-020-59596-x (PMC7026412; doi:10.1038/s41598-020-59596-x)
Supplement: Supplementary file 1 — Supplementary information [file 41598_2020_59596_MOESM1_ESM.docx]

***In vitro* replicative fitness of early Transmitted founder HIV-1 variants and sensitivity to Interferon alpha**

Manickam Ashokkumar^1,2*^, Aanand Sonawanne^1^, Maike Sperk^2^, Srikanth P Tripathy^1^, Ujjwal Neogi^2,3^ and Luke Elizabeth Hanna^1*^.

**Affiliations**

^1^Department of HIV/AIDS, National Institute for Research in Tuberculosis, Chennai, India.

^2^Division of Clinical Microbiology, Department of Laboratory Medicine, Karolinska Institute, Stockholm, Sweden.

*^3^*Department of Molecular Microbiology and Immunology, University of Missouri, Columbia, MO 65211, USA.

**File type**: Supplementary figures

*Corresponding authors

**Luke Elizabeth Hanna, PhD**

E-mail: [hannatrc@yahoo.com](mailto:hannatrc@yahoo.com)

**Manickam Ashokkumar, M. Tech**

E-mail: [ashokkumar.manickam.1@ki.se](mailto:ashokkumar.manickam.1@ki.se)

**
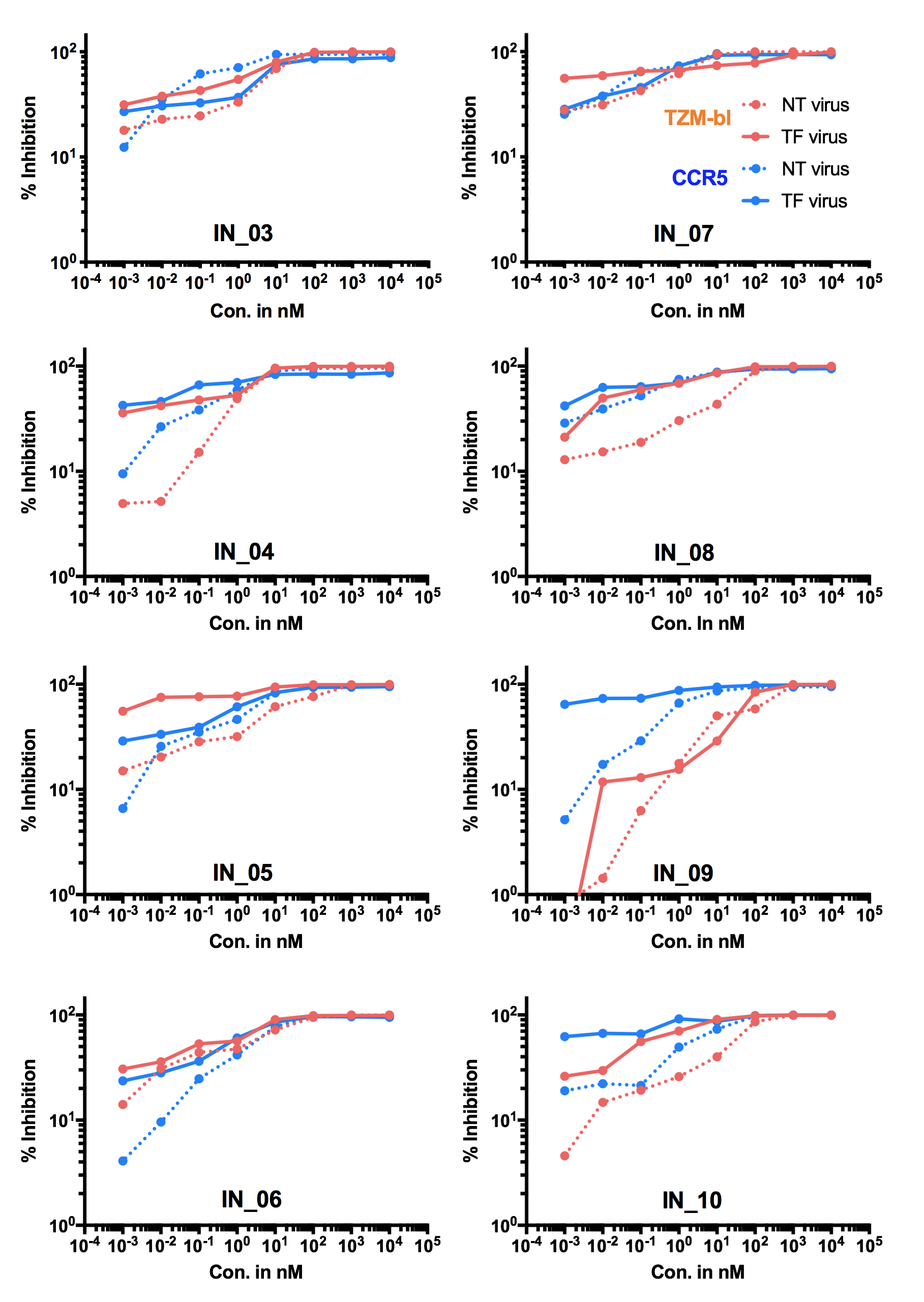
**

**Supplementary Figure 1.** Percentage inhibition of individual TF (solid line) and NT (dotted line) viruses by MVC in TZM-bl and GHOST (3) CCR5+ cells.

**
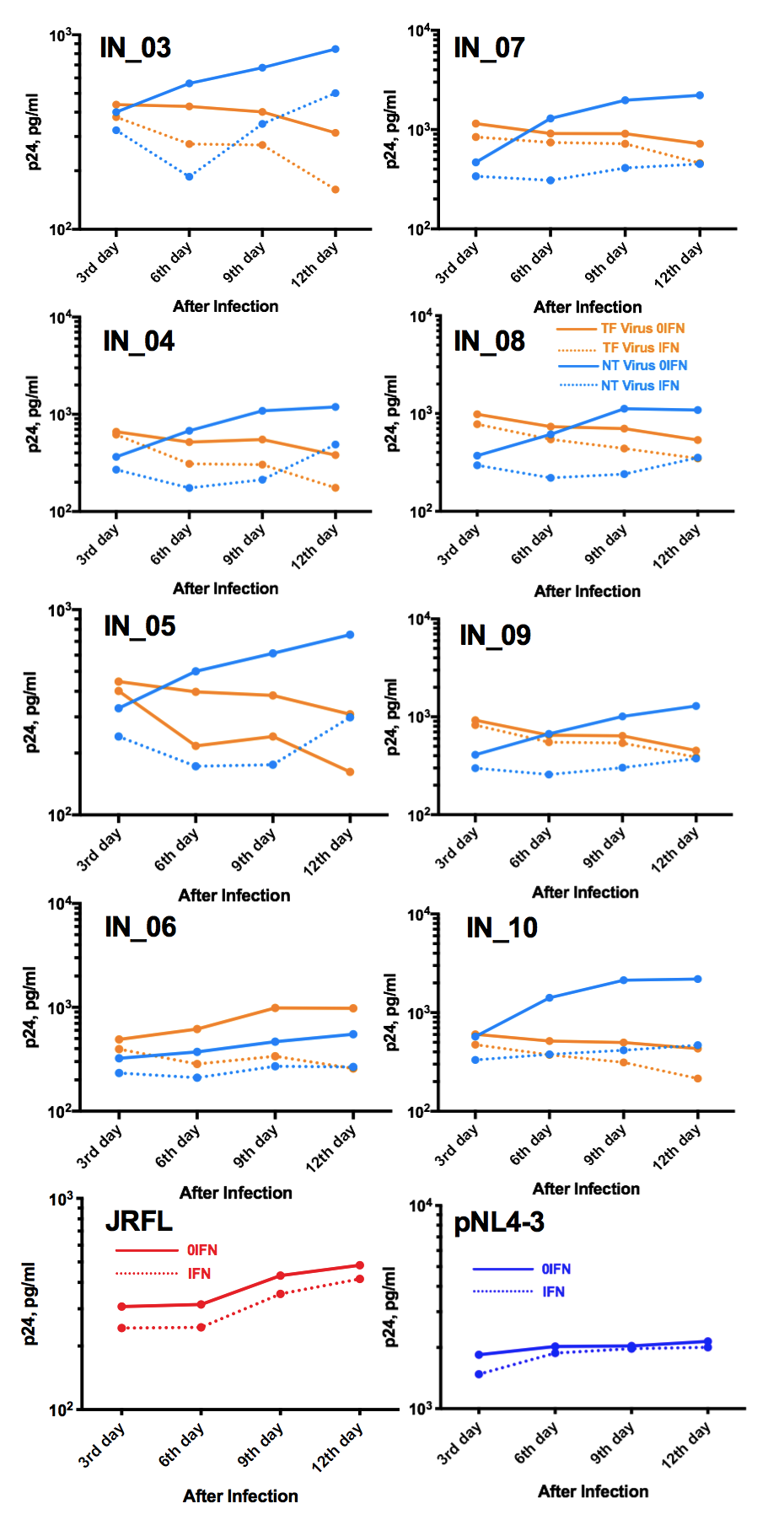
**

**Supplementary Figure 2.** Viral replication kinetics of individual TF and NT viruses including R5- and X4- tropic viral strains in primary CD4^+^ T cells.
